# A Five-Hole Pressure Probe Based on Integrated MEMS Fiber-Optic Fabry-Perot Sensors

**DOI:** 10.3390/mi15040554

**Published:** 2024-04-22

**Authors:** Yumiao Song, Shuanghui Ma, Jichun Zhao, Jia Liu, Jingyi Wang, Yongjun Cui

**Affiliations:** 1 State Key Laboratory of Dynamic Measurement Technology, North University of China, Taiyuan 030051, China; songym126@163.com (Y.S.); zhaojc1001@163.com (J.Z.); jialiu@nuc.edu.cn (J.L.); jingyiwang@st.nuc.edu.cn (J.W.); 2 Beijing Power Machinery Institute, Beijing 100074, China; mashuanghui@sina.cn

**Keywords:** five-hole probe, pressure measurement, fiber-optic Fabry–Perot, MEMS

## Abstract

The five-hole pressure probe based on Micro-Electro-Mechanical Systems (MEMS) technology is designed to meet the needs of engine inlet pressure measurement. The probe, including a pressure-sensitive detection unit and a five-hole probe encapsulation structure, combines the advantages of a five-hole probe with fiber optic sensing. The pressure-sensitive detection unit utilizes silicon-glass anodic bonding to achieve the integrated and batch-producible manufacturing of five pressure-sensitive Fabry–Perot (FP) cavities. The probe structure and parameters of the sensitive unit were optimized based on fluid and mechanical simulations. The non-scanning correlation demodulation technology was applied to extract specific cavity lengths from multiple interference surfaces. The sealing platform was established to analyze the sealing performance of the five-hole probe and the pressure-sensitive detection unit. The testing platform was established to test the pressure response characteristics of the probe. Experimental results indicate that the probe has good sealing performance between different air passages, making it suitable for detecting pressure from multiple directions. The pressure responses are linear within the range of 0–250 kPa, with the average pressure sensitivity of the five sensors ranging from 11.061 to 11.546 nm/kPa. The maximum non-linear error is ≤1.083%.

## 1. Introduction

The aerodynamic stability of an aero-engine is one of the important indexes for assessing the engine’s performance. With the integration of the inlet tract and the engine, the working conditions of the inlet tract are becoming more and more severe. Therefore, sensors need to be accurate in measuring the dynamic pressure in the high-speed flow field to accurately assess the impact of inlet pressure aberrations on the aerodynamic stability of the engine, and to provide a basis for the iterative optimization of the design of the inlet tract [1,2,3].

Five-hole probes [4] are typically used in three-dimensional, high-velocity flow field environments to obtain airflow velocity magnitude, direction, and static pressure. Most currently used probes are based on electronic pressure sensors [5,6,7]. Among them, long pressure line measurement leads to pressure loss and long response times. The piezoresistive sensors [8] on the probe surface affect the accuracy of the measurement results due to the introduction of new heat sources by the sensor. Compared with electronic pressure sensors, fiber-optic pressure sensors have the advantages of small size, anti-electromagnetic interference, high-temperature resistance [9,10], and high sensitivity [11], which can improve measurement accuracy. Pressure sensors are installed close to the probe tip, which leads to fast response times and high bandwidth [12]. Combined with MEMS technology [13], batch fabrication of the sensors can be achieved. Liu Yueying et al. proposed a differential fiber-optic airflow sensor based on a Fabry–Perot (FP) interferometer with a sensitivity of 826.975 nm/kPa and a resolution of 0.89 Pa in the measurement range of 0–11 kPa [14]. Chen Zhu et al. proposed a fiber-optic pressure sensor based on an optical fiber extrinsic Fabry–Perot interferometer (EFPI) with a sensitivity of 23.5 μm/kPa and a resolution of ±0.05 Pa [15]. Jia Liu et al. achieved the stable operation of a magnesium oxide FP pressure sensor at 22–800 °C and 0–0.6 MPa by changing the sensor material [16]. Zhou Haocheng et al. proposed a miniature five-hole probe based on a fiber-optic lever. The resulting five-hole probe has a fast response with a modeled settling time of 0.24 ms and a measured high-frequency range of about 3.5 kHz [17]. Currently, most studies on fiber-optic FP pressure sensors focus on single-point tests. Total pressure tube measurements can cause errors when assessing inlet distortion at large inlet outlet flow angles. Therefore, a multi-hole pressure probe is required for multi-directional pressure measurements to improve the inlet tract distortion assessment accuracy.

This study combined the advantages of optical fiber sensing and five-hole probes. A five-hole probe based on the optical fiber FP was proposed. The probe consists of a sensitive unit, a five-hole probe, and a base package structure. The structural parameters of the probe are optimized by fluid simulation. The pressure sensitivities of the sensitive unit are analyzed by mechanical simulation. The dimensions of the five-cavity integrated acute unit are determined by a combination of simulation and practice. Sensitive units with fixed cavity lengths are fabricated in bulk by dry etching and anodic bonding technology. The specific cavity length is extracted by using a non-scanning correlation demodulation instrument. A sealing experimental platform was built to verify that the five-hole probe and the five-cavity integrated sensitive unit have good sealing performance and can be used for accurate measurement of multi-directional pressure. A static pressure experimental test platform was built. The experimental results show that the response of each pressure cavity is linear. The average pressure sensitivities of the five sensors are 11.061–11.546 nm/kPa in the pressure range of 0–250 kPa and have good consistency. The maximum nonlinear error is ≤1.083%.

## 2. Design and Principle

### 2.1. Probe Design and Sensor Principle

**Figure 1:**
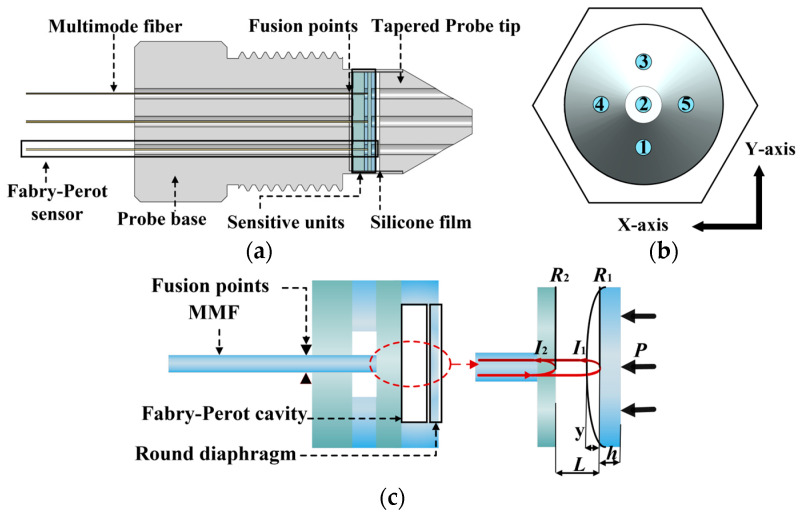
Schematic diagram of the five-hole probe and internal structure based on fiber-optic Fabry–Perot pressure sensors: (**a**) five-hole probe section; (**b**) top view of the five-hole probe; (**c**) schematic diagram of Fabry–Perot sensor and principle.

The design of the five-hole probe and its internal structure is shown in Figure 1a. The probe consists of a conical five-hole probe, a pressure-sensitive unit, a probe base, and five multimode mode fibers (MMF). The five-hole probe has five inlet holes for pressure conducting. The pressure-sensitive units are batch-prepared using the MEMS technology. Five cavities are processed on one pressure-sensitive unit. Integrating the sensitive units inside the probe reduces pressure conduction time. Figure 1b shows the top view of the probe. The holes along the *X*-axis are hole 5, hole 2, and hole 4 in sequence. The holes along the *Y*-axis are hole 1, hole 2, and hole 3 in sequence. Hole 2 is the central hole, while the others are edge holes. The probe base structure is designed with thread and hexagon structures for static pressure experiments. Each diaphragm’s deformation depends on the pressure conducted through the corresponding inlet hole, with a one-to-one correspondence between the FP sensor and the inlet holes in the probe structure.

The structure of one of the sensors inside the probe is schematically shown in Figure 1c. For Fabry–Perot structures, the variation in external pressure will result in the deformation of the membrane. The FP interference spectrum is shifted, and non-scanning correlation demodulation methods are employed to extract cavity length [18]. When a beam of light is injected into the FP cavity from the optical fiber, multiple reflections (*I*_1_ and *I*_2_) and transmissions occur on the surfaces of *R*_1_ and *R*_2_, leading to multiple-beam interference. The transmitted light undergoes multiple reflections on the surfaces of *R*_1_ and *R*_2_, forming a multi-beam interference pattern. The interference spectrum equation can be expressed as follows [19]:

(1) $$I=I_{1}+I_{2}+2\sqrt{I_{1}I_{2}}cos\left(\frac{4\pi nL}{\lambda^{2}}+\pi \right),$$

where *I*_1_ and *I*_2_ are the intensity of the reflected light on the *R*_1_ and *R*_2_ surfaces, respectively, *n* represents the air refractive index, *L* represents cavity length, and *λ* represents optical wavelength. According to elastic mechanics, the sensitivity of the circular diaphragm is obtained as follows [20,21]:

(2) $$S=\frac{y}{p}=\frac{3(1-\mu^{2})}{16Eh^{3}}R^{4},$$

where *y* is the deformation amount of the diaphragm subjected to pressure, *p* is the pressure exerted on the diaphragm, *E* is Young’s modulus, *μ* is Poisson’s ratio, and *h* and *R* represent the thickness and the radius of the diaphragm, respectively. When the diaphragm undergoes deformation within the elastic range, the maximum deformation of the diaphragm shall not exceed 30% of its thickness. The calculated maximum pressure measurement is:

(3) $$p_{max}=\frac{8Eh^{3}}{5(1-\mu^{2})R^{4}}.$$

### 2.2. Interrogation Method for FP Cavity Length

**Figure 2:**
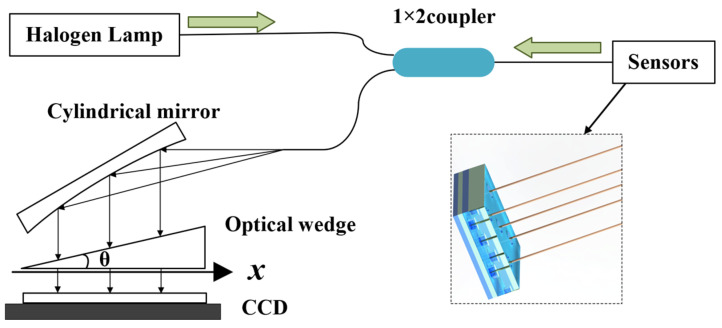
Schematic diagram of a non-scanning correlation demodulation system based on a broadband light source.

To measure a fixed air cavity length *L*, a non-scanning correlation demodulation system based on a broadband light source was established. The core idea of non-scanning correlation demodulation is the phase difference of light waves at different optical ranges. When a beam of light waves passes through a transparent film or other optical element, multiple beams of light are formed and have phase differences due to the different propagation speeds of the light. The system consists of a halogen lamp, a 1 × 2 coupler, a cylindrical mirror, an optical wedge consisting of two parallel glass plates, and a charge-coupled device (CCD), as shown in Figure 2. The halogen lamp emits a broadband light source that is directly coupled to the sensor via a 1 × 2 coupler. The reflected light carrying air cavity length information passes through the optical wedge. When the cavity length matches the thickness of the optical wedge, interference is formed on the surface of the optical wedge, and the demodulation of the cavity length is realized by using the cross-correlation operation [22]. Here, the optical wedge is designed for a thickness range of 8 to 22 μm.

The overall demodulation process is an optical correlation operation, and the outgoing light intensity after non-scanning correlation demodulation is as follows [23]:

(4) $$I_{out}=\int_{\lambda_{min}}^{\lambda_{max}}\frac{R_{1}+R_{2}+2\sqrt{{R_{1}R}_{2}}cos\left(\frac{4\pi L}{\lambda }\right)}{1+{R_{1}R}_{2}+2\sqrt{{R_{1}R}_{2}}cos\left(\frac{4\pi L}{\lambda }\right)}\cdot \frac{{\left(1-R_{3}\right)}^{3}}{1+R_{3}^{2}+2R_{3}cos\left(\frac{4\pi xtan\theta }{\lambda }\right)}\cdot I_{0}(\lambda )d\lambda .$$

where *λ_min_*~*λ_max_* is the wavelength range of the broadband light source, *I*_0_(λ) is the incident light intensity at wavelength *λ*, *R*_1_ and *R*_2_ are the end face reflectivity of the fiber optic Fabry–Perot pressure sensor, *R*_3_ is the reflectivity of the inner surface of the optical wedge, *x* is the position of a point on the optical wedge, *θ* is the angle of clamping of the optical wedge, and *L* is the length of the air cavity of the fiber optic Fabry–Perot sensor. The optical signal is converted to an electrical signal by signal processing using the CCD [24]. Through the inter-correlation operation of cavity length matching, the maximum value of optical signal intensity occurs at the position where the cavity length is the same as the thickness of the optical wedge. The peak search algorithm is utilized to obtain the location of the maximum value, and then the corresponding cavity length is calculated.

## 3. Simulation and Production

### 3.1. Probe Fluid Simulation

**Figure 3:**
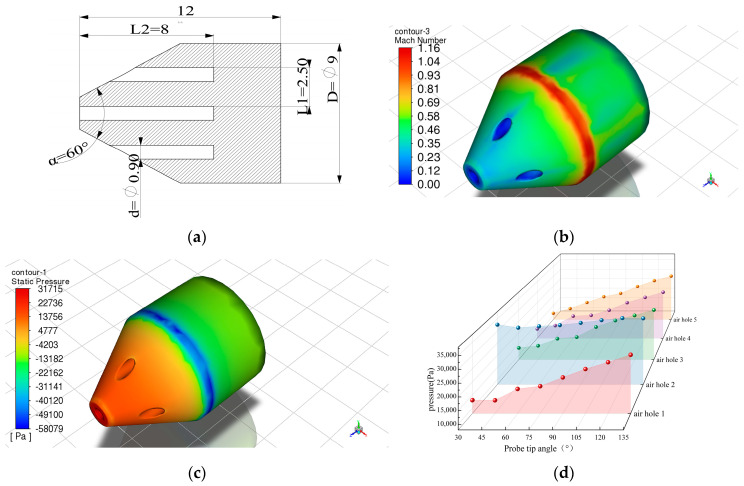
Simplified model of the probe and simulation results: (**a**) simplified model profile of the probe; (**b**) velocity cloud of the probe; (**c**) static pressure cloud of the probe; (**d**) the relationship between the cone angle at the probe tip and the pressure in each hole.

The probe was fluidly simulated to investigate the effect of structural parameters on the pressure transfer performance. The probe structure is shown in Figure 3a. The fluid Mach number was set to 0.6 Ma, and the grid turbulence model was set to the shear-stress transport (SST) k-omega model [25]. When the angle of incoming flow is 0°, the stationary point is formed at the center hole, and the center hole pore pressure is larger than the edge hole pressure. Multi-directional pressure measurement is achieved according to the pressure difference. According to the Bernoulli equation [26] and simulation results, it can be inferred that there is a ring of high-speed, low-pressure region in the intersecting contact area between the cone and the cylinder.

If the depth of the inlet hole is too small, the airflow has not reached a steady state, and the internal flow conditions cannot be accurately reflected [27]. If the depth of the inlet hole is too large, it leads to a longer time for the probe to guide the pressure. The aperture of the probe is related to the overall outer diameter of the probe and the diaphragm radius of the sensitive unit. A too-small aperture can cause blockages by small particles, affecting pressure measurement. A too-large aperture can increase the outer diameter of the probe, leading to greater interference in the flow field. The probe tip cone angle was gradually increased in 15° steps from 30° to 135°, while other structural parameters of the probe remained unchanged. When the inflow angle is 0°, fluid simulation is performed on the probe. Figure 3d shows the relationship between the probe tip cone angle and the pore pressure. When the probe faces the incoming flow, a stagnation point is formed at hole 2 of the probe. Due to the constant external Mach number, the static pressure at the center hole remains almost unchanged. As the cone angle parameter increases, while the depth of the center hole remains constant, the depth of the edge hole gradually increases, and the angle formed between the cone slope and the side becomes smaller. The static pressure of the edge hole gradually increases, the static pressure of the center hole slightly changes, and the difference between the center hole pressure and the edge hole pressure gradually decreases. Therefore, a probe with a large cone angle is suitable for precise measurements in environments with a low Mach number. A probe with a small cone angle is suitable for environments with a larger range of Mach numbers, making it more suitable for precise measurements of high-speed airflow pressure. The probe’s structural parameters are determined by the simulations and the actual conditions. The probe cone angle is 60°. The probe diameter is 9 mm, and the micro-hole diameter is 0.9 mm. The hole spacing is 2.4 mm, and the depth from the micro-hole to the sensitive diaphragm is 8.4 mm. Equalizing the radius of the sensitive diaphragm with the radius of the probe hole maximizes the effective diaphragm radius.

### 3.2. Mechanical Stimulation of Sensitive Unit

Considering the pressure measurement range of 0–250 kPa, the demodulation system, and the fabrication process, the final determination of the structural parameters for the sensitive unit was made. The diaphragm radius is 0.45 mm, the diaphragm thickness is 15 μm, and the cavity length is 15 μm. The maximum pressure measurement is 277.2 kPa. The theoretical pressure sensitivity is 16.232 nm/kPa. The overall dimensions of the sensitive unit are 6 mm × 6 mm × 2.03 mm. Simulations of the sensitive unit were conducted to obtain the simulated sensitivity of the sensor.

**Figure 4:**
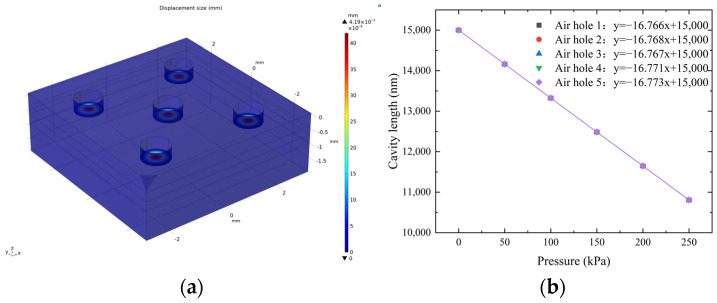
Simulation results for the sensitive unit: (**a**) diaphragm displacement cloud; (**b**) simulation results for sensitive unit sensitivity.

Finite element simulation software was used, the side of the sensitive unit was fixed, and a pressure of 250 kPa was applied to the diaphragm. The displacement cloud map of the sensitive unit was obtained, as shown in Figure 4a. With an increment of 50 kPa, simulations were conducted by gradually increasing the pressure from 0 kPa to 250 kPa to obtain the deformation values of five diaphragms. Combined with the initial cavity length, the simulated sensitivity of all five FP sensors was calculated to be 16.77 nm/kPa, as shown in Figure 4b.

### 3.3. Sensitive Unit Integrated MEMS Process Preparation

**Figure 5:**
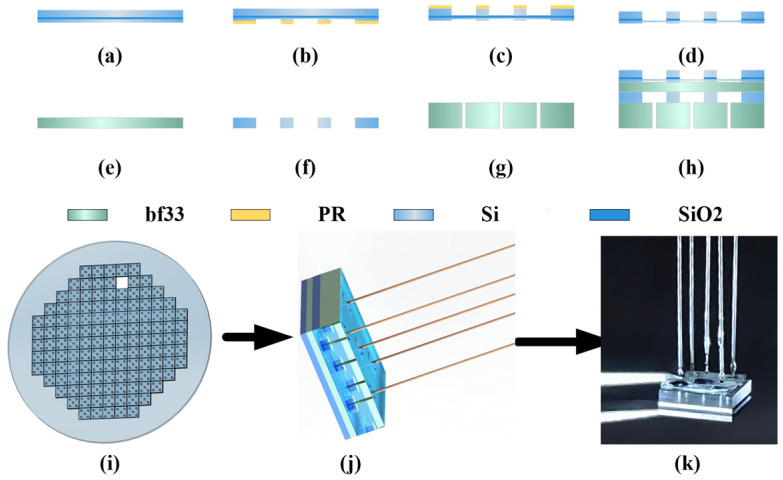
Sensitive unit MEMS process flow: (**a**) SOI wafer; (**b**) Frontside photolithography; (**c**) Backside photolithography; (**d**) Buried oxygen etching; (**e**) Glass wafer; (**f**) Perforated silicon wafers; (**g**) Perforated glass wafer; (**h**) Anodic bonding; (**i**) Integrated wafer; (**j**) Sensitive Unit schematic; (**k**) Physical image of sensitive unit.

The structure of the sensitive unit consists of a four-layer structure comprising etched silicon-on-insulator (SOI) wafers, glass sheets, perforated silicon, and perforated glass [28]. The process is divided into three main parts. The three main parts include the preparation of the sensitive diaphragm, integrated fabrication of the sensitive unit, and separation of the sensitive unit. The MEMS processing workflow is illustrated in Figure 5.

Firstly, the front-side processing of the SOI wafer (Figure 5a) involves photolithography and a deep-reactive ion etching (DRIE) step (Figure 5b). Back-side processing includes photolithography, DRIE, and reactive ion etching (RIE) steps (Figure 5c). The above process results in a cavity length and diaphragm thickness of 15 µm. The preparation of the sensitive diaphragm is completed. Secondly, perforated silicon wafers (Figure 5f) and perforated glass wafers (Figure 5g) are prepared using CNC technology. Anodic bonding is performed three times on the cleaned SOI wafer, glass wafer, perforated silicon wafer, and perforated glass wafer. The integrated fabrication of the sensitive unit is completed (Figure 5h). Finally, the four-layer integrated wafer (Figure 5i) is cut to obtain many sensitive units. The sensitive units are bonded to five multimode fibers (Figure 5g), and the physical representation is depicted in Figure 5k.

### 3.4. The Five-Hole Pressure Probe Preparation

**Figure 6:**
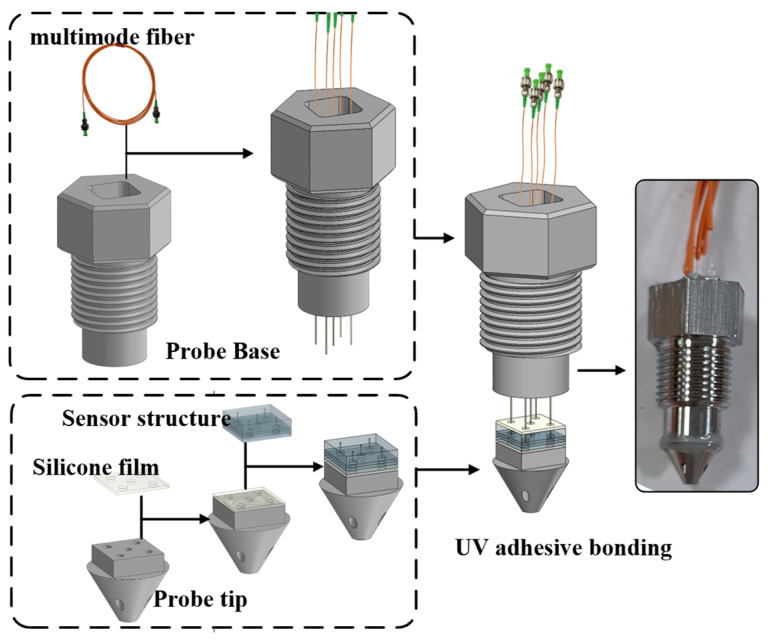
The five-hole pressure probe preparation process.

The preparation process for the five-hole pressure probe is divided into three steps, including the fixation of the five-hole probe and the sensitive unit, the fixation of the optical fiber and the sensitive unit, and the fixation of the five-hole probe and the probe base. The probe preparation process is illustrated in Figure 6.

Firstly, silicone gel is used as the bonding layer due to its buffering and sealing properties. Silicone gel is aligned and bonded to the structure of the five-hole probe based on the positions of the inlet holes. The probe tip is aligned with the sensitive unit and bonded. Secondly, silicone gel is used to align and bond to the sensitive unit based on the positions of the holes. A multimode fiber passes through the probe base. After aligning the optical fiber with the perforated glass structure, a high-temperature UV adhesive is applied. Finally, the probe tip is clamped onto the probe base. High-temperature-resistant adhesive is applied to the contact surface between the probe and the base. After waiting for the adhesive to fully cure, the probe base is injected with adhesive. The five-hole pressure probe is completed after adhesive solidification.

## 4. Experiments and Discussion

### 4.1. Sealing Test and Analysis

**Figure 7:**
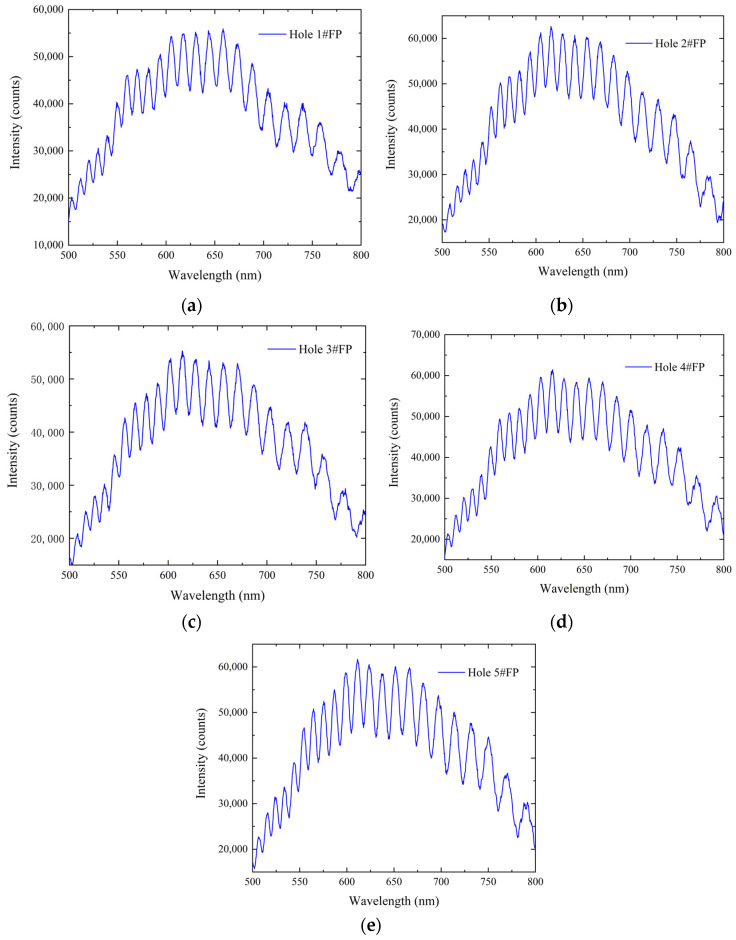
Initial spectra: (**a**) hole 1#FP spectrum; (**b**) hole 2#FP spectrum; (**c**) hole 3#FP spectrum; (**d**) hole 4#FP spectrum; (**e**) hole 5#FP spectrum.

The sensitive unit contains five diaphragm-type optical fiber FP pressure sensors. Before conducting experiments using probes, it is necessary to review the original spectral information of the five sensors. Spectral testing ensures that each sensor can be recognized by non-scanning correlated demodulators, thereby avoiding situations where the entire probe cannot be used due to the ineffectiveness of one sensor. The five fiber-optic Fabry–Perot sensors were numbered according to the location of the five holes of the probe, namely, hole 1#FP, hole 2#FP, hole 3#FP, hole 4#FP, and hole 5#FP. The raw spectral results for each sensor are shown in Figure 7. The signal strength is generally above 55,000, and the signal contrast is greater than 10,000, indicating that all five sensors have good signals and can be detected by the non-scanning correlation demodulator. The spectral differences between the five sensors are small, and the initial cavity lengths of the five sensors are close.

When airflow is introduced, each diaphragm in the five pressure sensors experiences pressure guided by its respective inlet hole. If the sealing of one of the inlet holes is compromised, it may affect other pressure sensors, leading to unreliable and inaccurate multi-directional pressure measurements. Therefore, before static pressure calibration, a sealing test must be conducted for each hole in the five-hole pressure probe.

**Figure 8:**
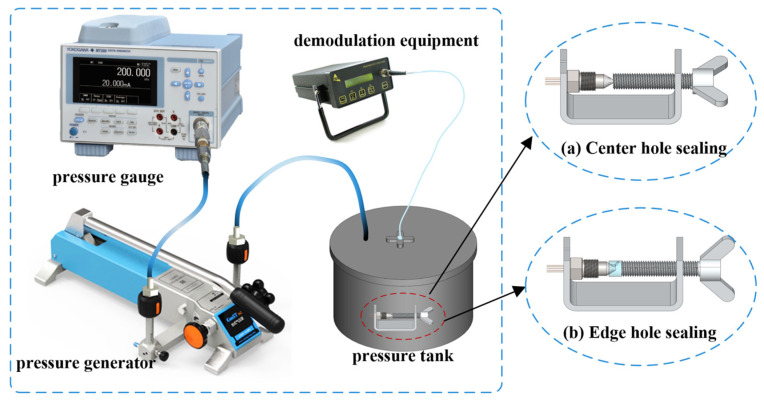
Sealing test platform: (**a**) center hole sealing; (**b**) edge hole sealing.

The sealed experimental platform consists of a pressure generator, a five-hole pressure probe, a pressure tank, a non-scanning correlation demodulator, and a high-precision pressure gauge, as shown in Figure 8. One end of the pressure generator is connected to the pressure tank, while the other end is connected to the high-precision pressure gauge. The pressure gauge displays the pressure generated by the pressure generator. The non-scanning correlation demodulator provides real-time displays of the cavity length. The probe is placed inside the pressure tank, and the signal is transmitted through the optical fiber to the non-scanning correlation demodulator. Due to the conical structure of the probe, the sealing test is divided into center hole testing and edge hole testing.

A U-shaped fixture with a 1 mm groove is designed for center hole sealing tests. The probe is placed in the middle and clamped to ensure that the pressure sensor corresponding to the center hole is not affected during the experiment (Figure 8a). For edge hole sealing tests, a 3D-printed structure is added between the U-shaped fixture and the probe. This structure matches the dimensions of the probe and has only one internal channel to ensure that only one hole is pressurized during the experiment (Figure 8b).

**Figure 9:**
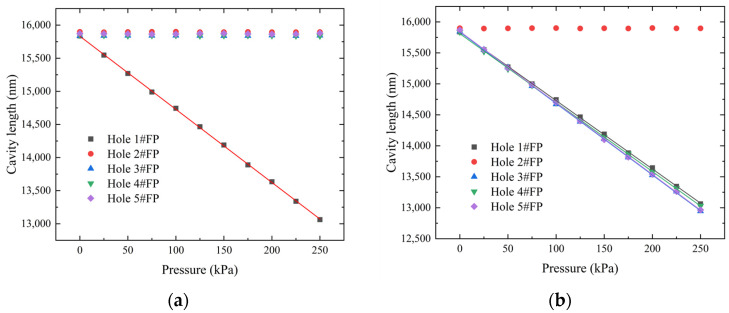

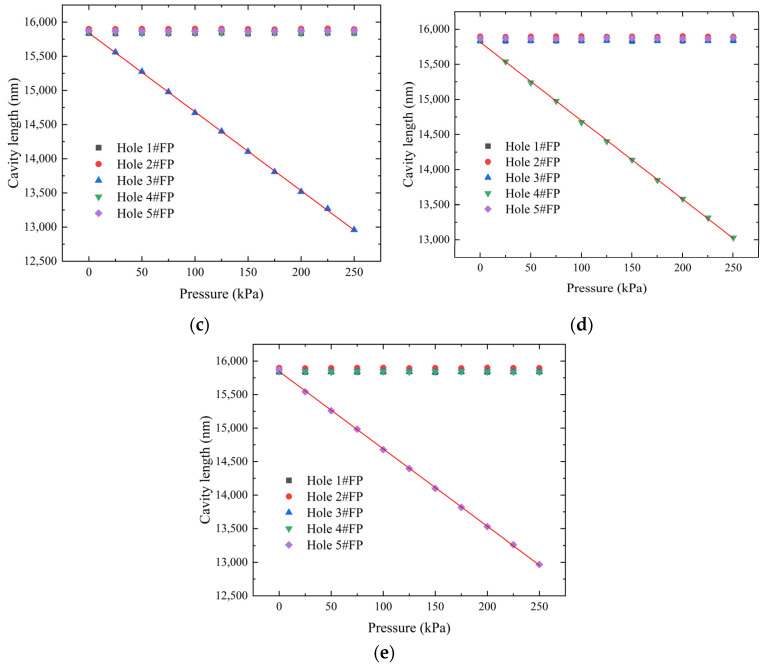
Sealing test results for inlet holes 1–5. Sealing results for (**a**) hole 1#FP; (**b**) hole 2#FP; (**c**) hole 3#FP; (**d**) hole 4#FP; and (**e**) hole 5#FP.

Sealing test results for the central hole and edge holes are shown in Figure 9. Taking edge hole 1 as an example (Figure 9a), when pressure is applied to edge hole 1, the cavity length of the corresponding pressure sensor shows a good linear relationship with pressure. The variations in cavity length for pressure sensors corresponding to other holes are within 20 nm, proving that hole 1 is sealed from other holes and the atmosphere. Figure 9b shows the sealing test results for the central hole. The pressure sensor corresponding to the central hole is not affected by pressure, proving that the central hole is sealed from each edge hole. The Fabry–Perot structure of the sensitive unit demonstrates good sealing with both the external environment and the probe. Each inlet hole can accurately transmit pressure to the corresponding diaphragm, enabling precise multi-directional pressure measurements.

### 4.2. Static Pressure Test and Analysis

**Figure 10:**
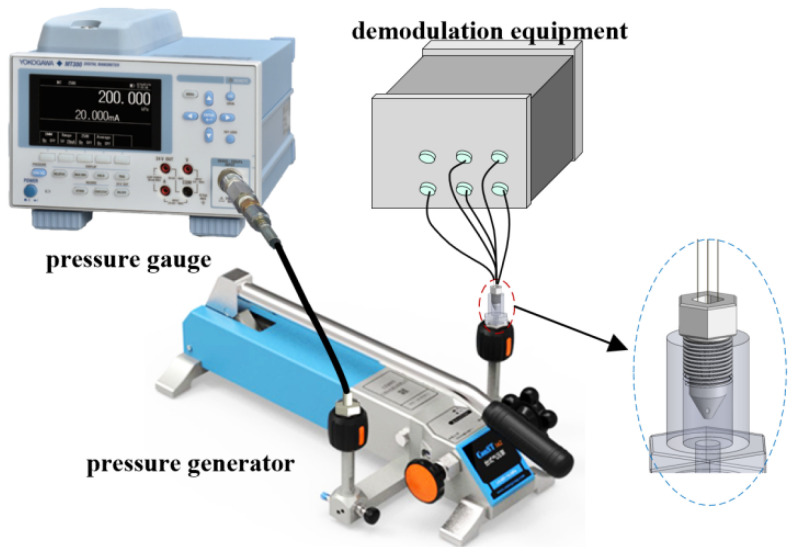
Schematic diagram of the static pressure test platform.

To simultaneously test the static pressure parameters of the five-hole probe, a static pressure testing platform was established, as shown in Figure 10. The platform consists of a pressure generator, a high-precision pressure gauge, a probe, and a non-scanning correlation demodulator. Repetitive pressurization and depressurization experiments were conducted within the range of 0–250 kPa at intervals of 25 kPa.

**Figure 11:**
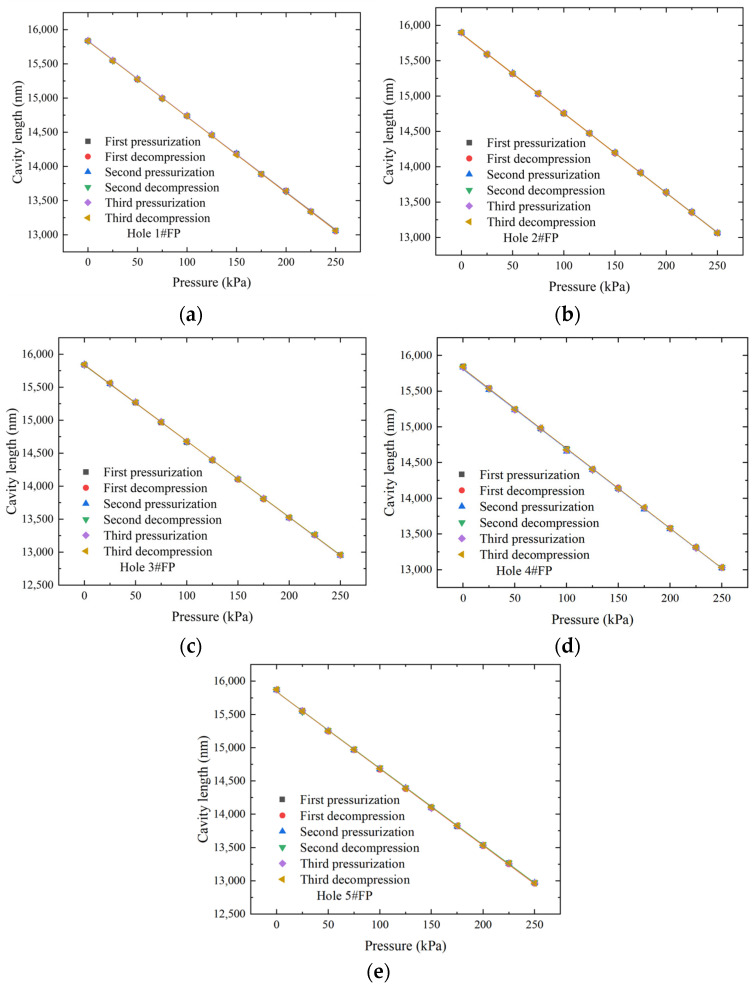
Five-hole probe hole 1–hole 5 static pressure test results for (**a**) hole 1#FP; (**b**) hole 2#FP; (**c**) hole 3#FP; (**d**) hole 4#FP; and (**e**) hole 5#FP.

The experimental results are shown in Figure 11, indicating a good linear relationship between the measured cavity length and pressure for each sensor within the range of 0–250 kPa. The performance parameters for each sensor were obtained through linear fitting of the data and error calculations, as shown in Table 1. The average static pressure sensitivity ranges from 11.061 to 11.546 nm/kPa. Along the *Y*-axis, the pressure sensitivity of hole 1#FP, hole 2#FP, and hole 3#FP increases sequentially. Along the *X*-axis, the pressure sensitivity of hole 5#FP, hole 2#FP, and hole 4#FP decreases in sequence. The main reasons for the actual pressure sensitivity being lower than the simulated sensitivity are twofold. Firstly, the center of the through-hole in the fourth layer does not coincide with the center of the diaphragm, resulting in a slight deviation between the center of the optical fiber and the center of the diaphragm. Secondly, the device layer thickness of the SOI wafer is 30 ± 1 μm, and there are variations in etching depth, leading to discrepancies between the actual diaphragm thickness and the designed diaphragm thickness.

## 5. Conclusions

For this study, we designed and fabricated a five-hole probe based on the MEMS technology. The probe primarily consists of a sensitive unit, a five-hole probe, and a base enclosure structure. Based on finite element simulation results and the actual conditions, the parameters for the pressure-sensitive unit and probe structure were determined. A batch production of five cavities integrated with a sensitive unit was carried out using the MEMS technology. A non-scanning correlation demodulation system was employed to demodulate specific cavity lengths. The sealing test verified the good sealing between the five-hole probe and the sensing unit. The probe performed multi-directional pressure detection. A static pressure test platform was established to analyze the pressure response characteristics of the probe. The experimental results showed that all five FP sensors exhibit good linear response in the 0–250 kPa range. The maximum nonlinearity error was ≤1.083%. For the five sensors, the maximum average pressure sensitivity was 11.546 nm/kPa, and the minimum average pressure sensitivity was 11.061 nm/kPa. The probe holds potential for applications in multi-directional pressure measurements in high-temperature environments. Further optimization of the sensitive unit fabrication process will aim to reduce the probe size and minimize airflow disturbances.

## Figures and Tables

**Table 1:** Performance parameters for the five-hole probe.

Performance	Hole 1#FP	Hole 2#FP	Hole 3#FP	Hole 4#FP	Hole 5#FP
Initial cavity length/nm	15,837	15,898	15,840	15,840	15,871
Average sensitivity/nm·kPa^−1^	11.061	11.256	11.546	11.166	11.528
Nonlinear error/%	0.789	0.701	0.792	1.083	1.058
Minimum R^2^	0.99979	0.99989	0.99986	0.99964	0.99979

## Data Availability

Data will be made available on request.
